# Phylogenetic and Taxonomic Status Analyses of the Abaso Section from Multiple Nuclear Genes and Plastid Fragments Reveal New Insights into the North America Origin of *Populus* (Salicaceae)

**DOI:** 10.3389/fpls.2016.02022

**Published:** 2017-01-04

**Authors:** Xia Liu, Zhaoshan Wang, Wenhao Shao, Zhanyang Ye, Jianguo Zhang

**Affiliations:** ^1^State Key Laboratory of Tree Genetics and Breeding, Key Laboratory of Silviculture of the State Forestry Administration, Research Institute of Forestry, Chinese Academy of ForestryBeijing, China; ^2^Collaborative Innovation Center of Sustainable Forestry in Southern China, Nanjing Forestry UniversityNanjing, China; ^3^Research Institute of Subtropical Forestry, Chinese academy of ForestryHangzhou, China

**Keywords:** plant evolution, phylogenetic, chloroplast DNA, single-copy nuclear, taxonomic status, *Populus mexicana*

## Abstract

Although, the Abaso section is widely accepted as an independent section, the taxonomic status of *Populus mexicana* (section Abaso) has not yet been resolved due to the limited availability markers and/or the lack of *P. mexicana* specimens in previous studies. Thirty-one poplar species that represent six sections of the *Populus* genus were sampled, and 23 single-copy nuclear DNA and 34 chloroplast fragments were sequenced. The present study obtained two updated phylogenies of *Populus*. We found that monophyly of the genus *Populus* is strongly supported by nuclear and plastid gene, which is consistent with previous studies. *P. mexicana*, diverged first in the nuclear DNA tree, which occupied the basal position, implying that the section Abaso may be the most ancestral lineage in extant populous species. Given that the short branches and low statistical support for the divergence of sections Abaso and Turanga, this observation probably indicated that a rapid radiation evolution following the early split of the genus *Populus*. In the plastid tree, *P. mexicana* clustered with modern-day species of section Tacamahaca in the plastid tree. Based on cytoplasmic and single-copy nuclear marker sequences, we hypothesized that chloroplast capture resulted in the inconsistent position of *P. mexicana* between the phylogenetic trees. Given the first unequivocal records of poplar fossils from the Eocene with similar leaf morphology to the extant *P. mexicana* and the phylogenetic positions of *P. mexicana* in our study, we support the hypothesis that the *Populus* genus originated in North America, which will provide new insights to the development of the origin of *Populus* species.

## Introduction

The genus *Populus* (Salicaceae), commonly known as poplars, are widely distributed in the northern hemisphere throughout the subtropical to boreal forests and are well-known for their economical, ecological and evolutionary importance as pioneer species (Stettler et al., 1996). According to a recent classification, the genus *Populus* is classified into 29 species in six sections (Abaso, Aigeiros, Leucoides, Populus, Tacamahaca, and Turanga) that are delineated by the occurrence of major hybridization barriers (Eckenwalder, 1996a). The phylogenetic analyses of the genus *Populus* have been performed using DNA sequences from both nuclear (nrDNA) and chloroplast (ptDNA) genomes (Leskinen and Alström-Rapaport, 1999; Azuma et al., 2000; Wang et al., 2014), and these analyses strongly suggest that *Populus* is a monophyletic group. However, these previous studies only focused on the five sections of the genus that excluded species of the section Abaso. Recently, a study by Cervera et al. (2005) covered six sections using 151 amplified fragment length polymorphism (AFLP) markers for discerning relationships among various poplar species, and their data suggested that *Populus mexicana* Wesmael (section Abaso) may be different from *Populus* or *Salix*, which is diametrically opposed to Eckenwalder’s (1996a) interpretation based on morphological traits. Thus, a few uncertainties and unanswered questions remain regarding the phylogenetic placement of section Abaso within the genus.

*Populus mexicana*, a rare tree, is the sole extant species of *Populus* L. sect. Abaso Eckenw. Although, *P. mexicana* is geographically restricted to the warmer areas of southern North America (NA) today, early fossil distributions reveal that ancestral poplars of section Abaso were widespread across NA and as far north as Alaska (Eckenwalder, 1977, 1980). The current populations generally comprise few individuals that are restricted to floodplains in Mexico (Eckenwalder, 1996b). Populations from humid eastern Mexico occupy a wide disjunctive region and usually contain fewer individuals than the fairly compact populations in the droughty northwestern region (Eckenwalder, 1996b). The extant *P. mexicana* leaves are remarkably similar to the earliest recognizable fossil species, *P. wilmattae* Cockrell (Manchester et al., 1986), which dates back to the Palaeocene, approximately 58 million years ago (Eckenwalder, 1996a). This information suggests that *P. mexicana* may be the most ancient species of *Populus*. The point of origin of Salicaceae has been more controversial. For example, the likely outgroups of Saliceae only are all extant in Asia today and not in NA, suggesting that the family originated in Asia, however, the occurrence of the earliest known fossils of both *Pseudosalix* (an extinct genus was proposed by Boucher et al., 2003) and *Populus* in the Eocene of Utah raises the possibility of an NA origin for Saliceae (Boucher et al., 2003). Moreover, as the discussion of origin and species taxonomy now relies solely on fossil records, much molecular evidence is required to support that speculation. *P. mexicana* is allopatric compared to all other NA species (Eckenwalder, 1996a) which have mostly parapatric distributions (Huang et al., 2014), and its crossing relationships are unclear (Eckenwalder, 1996a). Therefore, the molecular phylogenetic and cryptic origin study of *P. mexicana* will provide key clues as to the location of origin and evolutionary history of the *Populus* genus and NA poplar species.

The phylogenetic placement of *P. mexicana* has long puzzled systematists. Reconstructing the phylogenetic relationships using 23 single-copy biparentally inherited nuclear genes and 34 maternally inherited ptDNA fragments (Mejnartowicz, 1991) may shed light on the evolution of *P. mexicana* and the phylogenetic relationships related to other sections within the genus *Populus*. Therefore, the main goals of the present study were: (1) to determine the phylogenetic position of *P. mexicana*, and (2) to further explicitly investigate the origin of *P. mexicana* and its evolutionary relationships with other sections within the *Populus* genus as well as to further discuss the point of origin of the genus *Populus*.

## Materials and Methods

### Plant Materials

We sampled representative species of all six sections of the genus *Populus* following the taxonomy system of Eckenwalder (1996a) and the Flora of China (Wu, 1999). A total of 31 species representing all six sections of the genus *Populus*, and two *Salix* species, *Salix triandra* L. and *S. arbutifolia* (Pall.) A. Skv., as well as three species from three genera that are members of the Salicaceae family, *Idesia polycarpa* Maxim., *Itoa orientalis* Hemsl., and *Poliothyrsis sinensis* Oliv. were sampled as outgroups (Supplementary Table S1).

### DNA Extraction, PCR Amplification, and Sequencing

Total genomic DNA was isolated from fresh or silica-gel-dried leaves using a modified CTAB protocol (Doyle, 1987) and used as a template for the polymerase chain reaction (PCR). Twenty-three single-copy nuclear genes and 34 ptDNA fragments were used for amplification and sequencing. All the single-copy nuclear fragments were used in Du et al. (2014) except LX17 locus and LX20 locus, which were designed based on the genome sequence of *P. trichocarpa* T.& G. in this study. Twenty-two of the ptDNA fragments (beginning with YLT) were used in Wang et al. (2015), and the remaining 12 fragments were used in Wang et al. (2014). All primers used in this study are listed in Supplementary Table S2.

Polymerase chain reaction was performed using a programmable temperature gradient 96 U thermocycler (Applied Biosystems, Foster City, CA, USA). The reaction was carried out in a total volume of 30 μl containing 5–50 ng of DNA template, 3 μl of 10× buffer (Promega, USA), 2.4 μl of 2.5 mM of dNTPs, 2.4 μl of each prime(10 μM), and 0.15 μl of *Taq* DNA polymerase (5 U/μL, TaKaRa, Shiga, Japan). PCR amplification was performed using a gradient thermal cycler (Biometre or Eppendorf, Germany). The PCR cycle conditions were as follows: an initial denaturation step at 94°C for 4 min followed by 10 cycles of denaturation at 94°C for 30 s, an annealing step at 60°C for 30 s then 72°C for 2 min, followed by 26 cycles 94°C for 30 s, 50°C for 30 s then 72°C for 2 min and final extension at 72°C for 10 min. The PCR products were directly tested by 1.5% agarose gel electrophoresis to determine whether presence of the target fragments. After verification, the PCR products were bidirectional sequenced by Sangon Biotech in Shanghai using the same primers. If direct sequencing failed for certain samples, the purified PCR products were cloned into the pGEM – Teasy Vector System II (Promega, Madison, WI, USA). Six to fifteen positive clones of each sample were randomly picked and sequenced in both directions using the universal primer M13. All of the new sequences generated in this study have been submitted to GenBank; the accession numbers are listed in Supplementary Table S1.

### Data Analyses

Both the nuclear and chloroplast sequences were aligned using the program ClustalX (Thompson et al., 1997) and were refined manually in BioEdit (Hall, 1999). For all loci, regions with more than five mononucleotide or microsatellite repeats were excluded because of the uncertainty of homology that could be exacerbated by potential inaccuracies of the enzymatic process during PCR and sequencing (Kelchner, 2000; Zhu and Ge, 2005). Some loci or regions that failed to amplify and were treated as missing data in the subsequent phylogenetic analyses. Phylogenetic relationships were reconstructed with maximum parsimony (MP), maximum likelihood (ML), and Bayesian inference (BI) using PAUP/4.0b10 (Swofford, 2003), PhyML-v2.4.4 (Guindon and Gascuel, 2003) and MrBayes 3.1.2 (Ronquist and Huelsenbeck, 2003), respectively, with *S. triandra, S. arbutifolia, Idesia polycarpa, Itoa orientalis*, and *Poliothyrsis sinensis* as outgroups.

For the MP analysis, all characters were treated as unordered and equally weighted. A heuristic search was performed with 1000 replicates of random stepwise addition of sequences and the number of trees held in RAM was set to be 100000, with tree-bisection–reconnection (TBR) branch swapping and MULTREES on. The bootstrap analysis was conducted with 1000 replicates using simple taxon addition (Felsenstein, 1985).

The evolutionary models for the ML and BI phylogenetic analyses were determined by jModelTest 2.1.1 (Posada, 2008) with an Aikaike Information Criterion (AICc). The variability of alleles from each individual and the sequence characteristics of each nuclear gene and plastid gene as well as the most appropriate models fitting each locus are shown in Supplementary Table S3. ML analyses for heuristic tree searches were carried out with the selected substitution model, random taxon addition of 1000 replicates, TBR branch swapping, the MULPARS option on, 100000 trees held in RAM and 100 replications of the bootstrap analysis. For BI, we conducted two independent Markov Chain Monte Carlo (MCMC) runs, each consisting of one cold and three heated MCMC chains that were run for 1000,000 generations and sampled every 100 generations; all other parameters were set to default. The first 25% of the sampled trees were discarded as burn-in to ensure that the chains had become stationary, and the posterior probabilities were calculated from the remaining trees. The statistical support values were presented on the phylogenetic trees. All the phylogenetic trees were viewed in the program FigTree v 1.4.2 (Rambaut, 2014).

## Results

### Sequence Characteristics

Twenty-three single-copy nuclear DNA gene and 34 plastid fragments were all successfully amplified and directly sequenced for all samples. After removing regions with mononucleotide repeats and microsatellite sequences, the aligned lengths of the nuclear DNAs ranged from 233 bp (locus LX17) to 1109 bp (locus DSH10), with a total length of 14851 bp; the number of variable sites ranged from 46 (locus DSH6) to 175 (locus DSH10) and that of informative sites ranged from 37 (locus DSH6) to 137 (locus DSH22). The aligned lengths of plastid fragments varied between 564 and 2601 bp with a total length of 36031 bp, which corresponds to 23.0% coverage of the whole chloroplast genome of *P. trichocarpa* T.& G. (Tuskan et al., 2006); the total number of variable sites was 850, of which 302 were parsimony informative.

### Phylogenetic Analysis

#### Nuclear Gene Phylogenies

**FIGURE 1:**
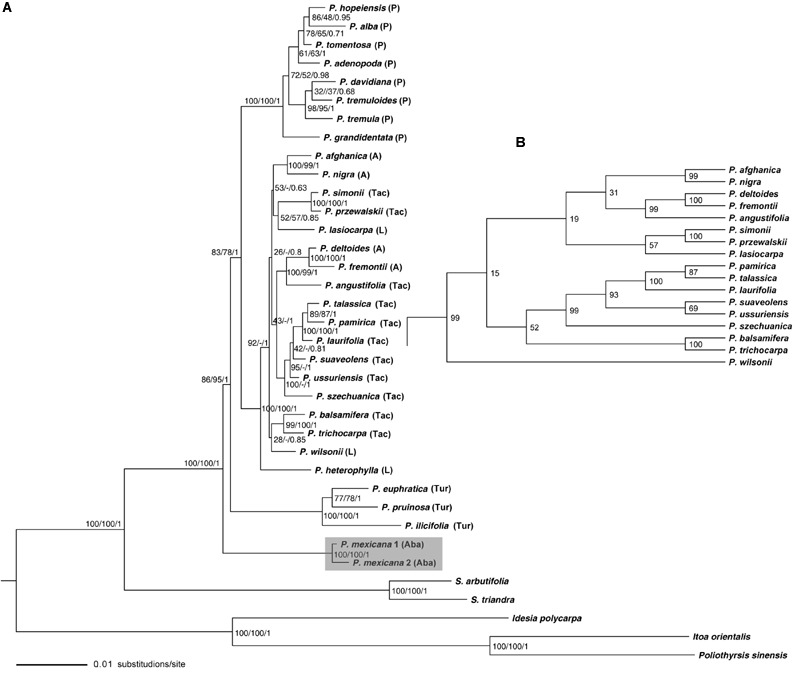
**Phylogeny of *Populus* inferred from the combined 23 single-copy nuclear DNA sequence using ML method. (A)** Numbers near the nodes sequentially indicated ML/MP/BI support values. **(B)** Indicates the topology difference derived from MP analysis. P, Populus; A, Aigeiros; L, Leucoides; Aba, Abaso; Tac, Tacamahaca; Tur, Turanga.

The best fitting evolutionary model for the combined 23 nuclear DNA data set was TIM2+I+G in the ML and Bayesian analyses. The most parsimonious (MP) tree, tree length = 4606, consistency index (CI) = 0.809, retention index (RI) = 0.805, and rescaled consistency index (RC) = 0.651, and the ML trees were generated from the two most parsimonious trees and the six most likely trees, respectively, based on the combined nuclear DNA data set. The nuclear gene phylogenetic trees generated from the MP, ML and Bayesian methods had identical topology to each other with only a few differences in bootstrap support (BS) or posterior probability (PP) values in some clades (**Figures 1A,B**).

**FIGURE 2:**
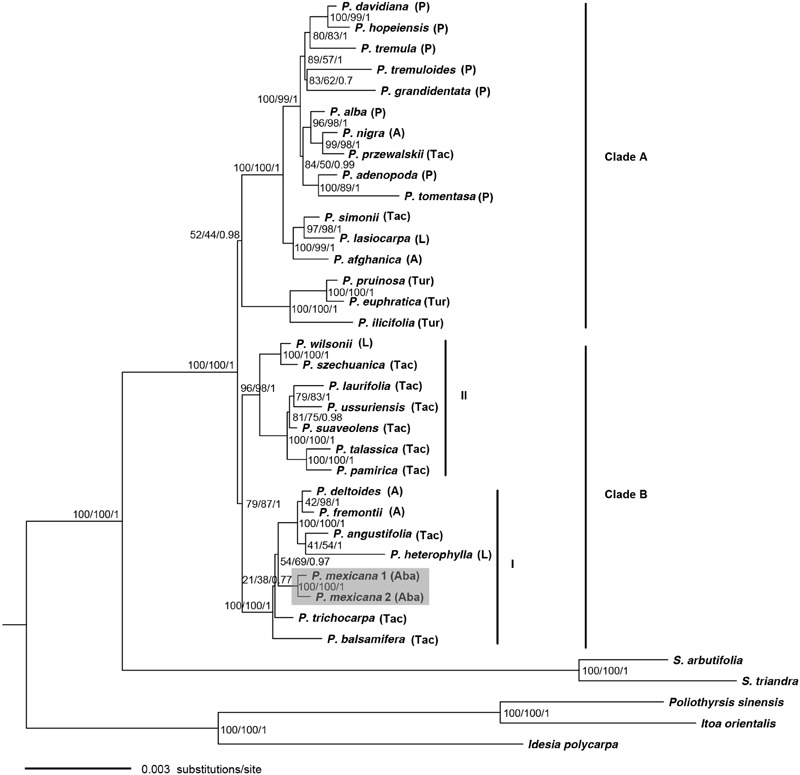
**Phylogeny of *Populus* inferred from the combined 34 plastid fragments using ML method.** Numbers near the nodes sequentially indicated ML/MP/BI support values.

In the nuclear phylogeny (**Figure 1A**), monophyly of *Populus* was strongly supported. *P. mexicana* of section Abaso diverged first with high support value, in a basal position; followed by section Turanga. The species of section *Populus* formed its own clade. For the remaining species from Tacamahaca, Leucoides, and Aigeiros sections clustered together as one clade with high resolution. The support values for the divergence of the first two sections Abaso and Turanga are slight low compared to the support for the monophyly of the whole genus *Populus* or for the section *Populus* or for the complex Aigeiros/Tacamahaca/Leucoides, which might be an indication for a rapid radiation without a very clear order of divergence. In the complex Aigeiros/Tacamahaca/Leucoides clade, *P. heterophylla* L. of section Leucoides was sister to other species. *P. fremontii* S. Watson of Aigeiros and *P. angustifolia* James of Tacamahaca show close affinity to each other (**Figures 1** and **2**). Aigeiros section poplars and balsam poplars are frequently interfertile. Evidence for ongoing introgressive hybridization has been found in *P. fremontii* and *P. angustifolia* (Keim et al., 1989), and our results also detected this pattern.

#### Chloroplast DNA Phylogenies

Phylogenetic trees generated by single chloroplast fragments were similar because all plastid gene sequences are effectively inherited as one locus in plants. Thirty-four plastid gene sequences were concatenated into a single contiguous sequence for the phylogenetic analysis. The best fitting evolutionary model for the combined plastid data set was TVM+I+G in the ML and Bayesian analyses. The MP tree, tree length = 2893, consistency index (CI) = 0.878, retention index (RI) = 0.868, and rescaled consistency index (RC) = 0.762, and ML trees were generated from the six most parsimonious trees and the seven most likely trees based on the combined plastid data set. The phylogenetic trees generated by the parsimony, ML and Bayesian methods were most similar to each other but with different branch support in some clades (**Figure 2**).

In the ptDNA phylogeny (**Figure 2**), the monophyletic relationships of *Populus* were also strongly supported. However, in contrast to the nuclear gene phylogeny, the species of the *Populus* genus were divided into two strongly supported clades corresponding largely to geographic distributions (**Figure 2**). Clade A comprised all Eurasian species except two NA species *P. tremuloides* Michaux and *P. grandidentata* Michaux as well as *P. ilicifolia* Rouleau in Turanga lineage from Africa. Clade B was further divided into two subclades (I and II). Subclade I consisted of all the NA species (*P. mexicana, P. balsamifera* L., *P. trichocarpa, P. heterophylla, P. angustifolia, P. fremontii*, and *P. deltoides* Marshall) and was clustered together sister to subclade II, which consisted of seven Eurasian species.

## Discussion

### The Phylogenetic Position of *P. mexicana*

In molecular phylogenetic analyses of closely related species, a combined analysis of multiple loci (Levsen et al., 2012; Zhou et al., 2014; Brassac and Blattner, 2015; Hao et al., 2015) is needed to overcome the stochastic variation inherent from locus to locus and to accurately reconstruct phylogenetic relationships of taxa (Wendel and Doyle, 1998). With a large combined data set of 23 single-copy nuclear DNA and 34 ptDNA fragments, we reconstructed phylogenies of *Populus* (**Figures 1** and **2**, respectively). All species of *Populus* clustered together as a well-supported clade separated from the outgroup species that was consistent with previous studies which supported the monophyletic nature of *Populus* (Hamzeh et al., 2006; Wang et al., 2014). However, the taxonomic status of *P. mexicana* has been disputed in previous phylogenetic studies (Eckenwalder, 1996a; Cervera et al., 2005), and the exact systematic position remains unclear. For example, a phylogenetic analysis based on the AFLP sequence suggested that the position of *P. mexicana* is located outside from the outgroup of several *Salix* species, while section *Populus* was the oldest lineage in *Populus* (Cervera et al., 2005). Eckenwalder (1996a) suggested that sections Abaso and Turanga are sister to the other sections of *Populus* based on morphological phylogenetic analysis. Manchester et al. (1986) report fossil from the Eocene with similar leaf morphology of the extant *P. mexicana*, which showed that *P. mexicana* may be an ancestral lineage of *Populus*. In our nuclear phylogeny, *P. mexicana* diverged first and formed a single clade with high support, and the support value includes not only the most recent common ancestor (MRCA) of all poplars, but also the support value for the MRCA of all poplars except sect. Abaso (**Figure 1**). Given that biparentally inherited nuclear gene loci are more effective for species delimitation than maternally inherited ptDNA markers in many angiosperm genera (Bai et al., 2010; Kikuchi et al., 2010), and the extant *P. mexicana* species are morphologically distinct and reproductively isolated from other NA species (Eckenwalder, 1996a), it is quite reasonable that *P. mexicana* would form its own section. In addition, the placement of *P. mexicana* in a basal position of the genus *Populus*, which is consistent with Eckenwalder’s interpretation based on morphological traits, the leaf morphology of extant *P. mexicana* was similar to the oldest poplar fossil from the Eocene (Manchester et al., 1986) together suggested that section Abaso may be an ancestral lineage.

In the present study, the larger data set was used to reconstruct a phylogenetic tree, in which the phylogenetic relationships among six sections were largely resolved (**Figure 1**). However, it is noteworthy that the internal branches of the basal lineages between the MRCA nodes of all poplars to MRCA node of all poplars except section Abaso were very short (**Figure 1**). Given that rapid speciation or radiation evolution is often featured by short internal branches in phylogenetic trees (Fishbein et al., 2001; Verboom et al., 2003; Whitfield and Lockhart, 2007; Zou et al., 2008). This observation probably indicated that the basal lineages between section Abaso and section Turanga radiated in rapid succession following the early split of the genus *Populus* could not be excluded entirely. A detailed study sampled more species and even genome data would be explained the result of rapid radiation evolution involving these lineages in the furfure.

### Incongruence Between Nuclear and Chloroplast DNA Phylogenies in the Placement of *P. mexicana* : Chloroplast Capture

Incongruence between phylogenetic trees based on nuclear and chloroplast marker sequences are commonly caused by incomplete lineage sorting, convergent evolution or hybridization and introgression(Degnan and Rosenberg, 2009; Acosta and Premoli, 2010; Pelser et al., 2010). Sequence convergence evolution would be highly unlikely because many of the observed mutations arose within non-coding regions. The time required for lineage sorting, they are comparatively short in evolutionary timescale of a genus. Lineage sorting of tree species would have taken millions of years to reach reciprocally monophyly of ptDNA for some tree species, and this stochastic process would not be expected to show the strong geographical partitioning observed here (**Figure 2**). Chloroplast capture, the introgression of a chloroplast genome from one plant species into another following a hybridization or ongoing backcrossing of F1s with parental populations, has frequently been thought to explain the inconsistencies between gene trees based on nuclear and cytoplasmic markers in plants (Smith and Sytsma, 1990; Rieseberg and Soltis, 1991; Tsitrone et al., 2003; Acosta and Premoli, 2010). A most remarkable outcome of chloroplast capture is that plastid phylogenies are often associated with geographic partitioning rather than with taxonomic relationships (Acosta and Premoli, 2010). *P. mexicana* showed close genetic affinity to Tacamahaca section species with high BS or PP value based on the ptDNA phylogeny (**Figure 2**), while *P. mexicana* occupied the basal position in the nuclear gene tree (**Figure 1A**).

**FIGURE 3:**
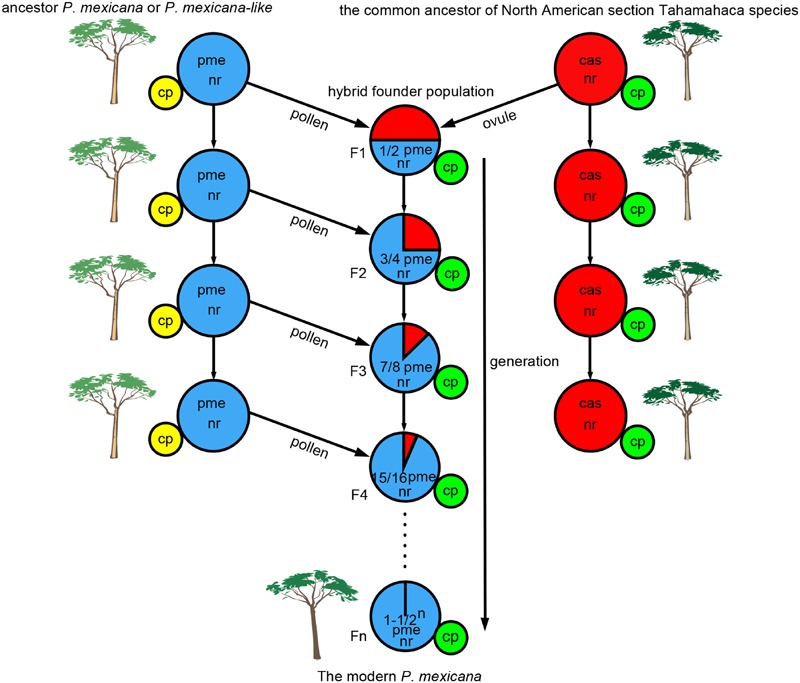
**A hypothetical scenario for chloroplast captures in ancient *Populus mexicana* and the common ancestor of North American section Tahamahaca species.** pme, *P. mexicana*; cas, the common ancestor of North American section Tahamahaca species; nr, nuclear DNA; cp, cytoplasmic DNA.

Chloroplast capture could occur frequently in species with sympatric distribution or contact zones and reproductive compatibility (Acosta and Premoli, 2010). According to fossil data, an ancestor of *P. mexicana* or *P. mexicana*-like was previously found in northern NA as far north as Alaska (Eckenwalder, 1977, 1980). *P. balsamifera* and *P. trichocarpa* in section Tacamahaca are distributed across northern NA, from Alaska to Newfoundland (Huang et al., 2014), and are distributed throughout NA western of the Rockies, ranging as far south as California and as far north to Alaska (Slavov et al., 2012), respectively. This suggests that the distribution of ancient *P. mexicana* and *P. trichocarpa* as well as *P. balsamifera* were likely to have had extensive overlap in geological history. The common ancestor of North American section Tahamahaca species received pollen from paternal parent species (*P. mexicana* ancestor or *P. mexicana*-like, which is now an extinct taxon), and such progeny could become hybrid founder populations. Since then, continual backcrossing with *P. mexicana* ancestor populations led to nuclear genes that were mostly from ancient *P. mexicana*, but ptDNAs from the common ancestor of North American section Tahamahaca species were preserved (**Figure 3**). As above discussion, we infer that the ptDNA gene from the common ancestor of North American section Tahamahaca species may be have conferred a selective advantage when ancient *P. mexicana* shifted southward and resulted in long-term adaptive evolution in Mexico because the global climate cooling (Hewitt, 1996; Comes and Kadereit, 1998; Hewitt, 2000; Barnosky et al., 2005). Of course, further studies, especially the analysis of maternally inherited plastid DNA and the utilization of additional gene markers or even genomic data, are needed to shed light on the complicated origin and evolution history of *P. mexicana* species.

### Evolutionary History of *P. mexicana* and Implications for the Origin of the *Populus* Genus

The exact location of the origin of the genus *Populus* remains unclear (Du et al., 2015), although large-scale morphological and molecular phylogenetic analyses of the *Populus* genus have been conducted in previous studies. Currently, there are two main hypotheses about the origin of the genus. The present study supports the NA origin hypothesis, given that *P. mexicana* occupied a basal position in the nuclear DNA tree (**Figure 1A**), which is consistent with Eckenwalder’s (1996a) investigations.

In fact, our results are also congruent with the paleontological evidence. The oldest fossil species (*P. wilmattae*), which occurred in northeastern Utah (USA) in the Middle Eocene (Manchester et al., 1986), is most similar to modern *P. mexicana*. Many poplar leaves and pollen fossils have been reported from the Eocene sediments of NA (Manchester et al., 1986, 2006; Boucher et al., 2003), which predates any fossil records in Asia (Du et al., 2015). The modern Turanga lineage is also likely an ancestral lineage based on heteroblastic characteristic, with willow-like juvenile leaves and tri-valvate capsules strongly differentiating it from other *Populus* species. That is most likely because the two sections diverged from a common ancestor.

Therefore, we speculate that a common ancestor of the *Populus* species first appeared on the NA continent and was then dispersed to other continents via the North Atlantic Land Bridge (NALB) and the Bering Land Bridge (BLB). Indeed, the two land bridges were present for the migration of the flora between Eurasia and NA (Tiffney and Manchester, 2001; Milne, 2006). Afterward, the breakup of the two epicontinental seaways resulted in divergence of numerous species because of vicariance (Li et al., 2013). Although the most extant diversity of poplar is in Asia, the center of diversity is not necessarily the place of origin that has been established by many plant molecular phylogenies (Ran et al., 2006). The eastern Asia has higher diversity (comparing to NA), it is possible that topographical heterogeneity which act both as museum and cradle for high plant species diversity in eastern Asia, and extinction of many NA species may also have had a role in the diversity bias favoring eastern Asia (Qian and Ricklefs, 2000). Furthermore, taxonomists have split Asian disjunct species more than those in North America by applying names to poorly differentiated allopatric populations (Qian and Ricklefs, 2000), that is, the application of the different species concept. To a certain extent, lumpers in NA may recognize additional geographical subdivisions of the species at the subspecies and variety levels that are treated as species by splitters in Asia (Eckenwalder, 1996a) could not be also excluded entirely. Further studies using more gene markers, or even genomic data, and more individuals per species should be pursued to untangle the mystery of the origin of *Populus* species in the future.

## Author Contributions

In this research, JZ and ZW were responsible for the experimental conceive and design. XL performed most of the experiments and drafted the manuscript. XL and ZW analysed the data, revising and finalizing the manuscript. XL, ZW, WS, and ZY were contributed reagents/materials/analysis tools. All the authors in this research read and approved the final manuscript.

## Conflict of Interest Statement

The authors declare that the research was conducted in the absence of any commercial or financial relationships that could be construed as a potential conflict of interest.
