# Supplementary material for: Phylogenetic and Taxonomic Status Analyses of the Abaso Section from Multiple Nuclear Genes and Plastid Fragments Reveal New Insights into the North America Origin of Populus (Salicaceae)
Source: Front Plant Sci. 2017 Jan 4;7:2022. doi: 10.3389/fpls.2016.02022 (PMC5209371; doi:10.3389/fpls.2016.02022)
Supplement: Supplementary file 2 [file Table_2.DOCX]

Supplementary Material

Phylogenetic and Taxonomic Status Analyses of the Abaso section in *Populus* from Multiple Chloroplast and Nuclear DNAs reveals new insights into the North America origin of *Populus*

Xia Liu*, Zhaoshan Wang, Wenhao Shao, Zhanyang Ye, Jianguo Zhang

*** Correspondence:** Xia Liu: liuxiavip8@163.com

# Supplementary Tables

**Table S2. The information of primers used in this study.**

| **Nuclear**  **Locus** | **Forward (5’-3’)** | **Reverse (5’-3’)** | **Annealing temperature（℃）** |
| --- | --- | --- | --- |
| DSH1 | ATTGAGGCTTTTGTTCAGCGGTTAT | CCTGTACTTGTTTGTCTGGCTTTGT | 58 |
| DSH2 | CATCTTTTGCCTTATTGTCTGCT | TGCGTTAAATGATCTTTCTGGTA | 56 |
| DSH3 | TCTGCTTTCCACTTCTTGC | CATACTCTCCCATTGTCCC | 55 |
| DSH4 | CCACCGCTACTCCTCCG | TCCACCCCTCCATCCAC | 58 |
| DSH5 | TGGCAGAATCACCAGACCCTC | CCAATTTAGCATCTTCAGCCTCAT | 59 |
| DSH6 | GCCTCCTGATTATTATGC | TATTACAAGCCCTTCCAG | 54 |
| DSH7 | TGTCCACAAACGCATCC | CAAACTTTACCACCCCA | 58 |
| DSH8 | GTTTGTTGTTCTGTTGATTGT | GGCTTCTCTTCTCTGATATTT | 56 |
| DSH10 | TACAAAAGCATTAAAGATCACCACT | GGAGCACACTTATCAATAAAACTAC | 54 |
| DSH12 | CACCACATCCCGCTTTCTCTCTTCACTT | TAAACCCCAGGAGGCAAAACAGCACCAG | 57 |
| DSH14 | TGTTTGATGGACCTGGCTGCT | CGGTTTATTGCCTTGTGGAGA | 55 |
| DSH15 | CTGAAAGGGAAAATAGTGGACAGTCAA | GGATAACAGTAGCATGGAGATATGGAT | 56 |
| DSH19 | AAGTCTGGTCAAGGCAGTGGTC | TCTGTGCTGTGATGTTTGGGGG | 54 |
| DSH21 | CATGCTTATGAAGGTGTGGGCTT | TGCAAACATCTCACTGGTGACTG | 53 |
| DSH22 | GTTGATTGGGCAAAAGAGG | ACAGCACGGTCCTTGGTTA | 58 |
| DSH23 | TATTCCTGCTGCTTACCTGAG | TGTGCTTCATCAATAACAATC | 55 |
| DSH24 | AAAACTGGAAAGAACTACTG | ATAATAACCTCGCTCACAAT | 56 |
| DSH25 | GCTCAAGCGATCATTCACA | TTTGCCAGAGTAGCCACGA | 54 |
| DSH26 | GGCTACTCTGAGACCCACTG | GCCATCAATTCCACCAACTA | 57 |
| DSH27 | ACAAGGAGGAAATAAATCAG | GCAGAGCAACAGGGAAAA | 53 |
| DSH29 | TATGGAAAAAGTTATGCCAAGAGGA | CAAAGGAGCAGAAGGCTATATCAAG | 54 |
| LX17 | GAGAAGCCAGCTTCTGTCT | CTAGCTTGTGGACAAGCTT | 58 |
| LX20 | GTGAGATAGATCAAGTGCAG | ACGTCCGCGACTCCGAGA | 59 |
| **Plastid locus** |  |  |  |
| *trnL-trnF* | CGAAATTGGTAGACGCTACG | ATTTGAACTGGTGACACGAG | 54 |
| *trnT-trnL* | CATTACAAATGCGATGCTCT | TCTACCGATTTCGCCATATC | 56 |
| *rbcL* | ATGTCACCACAAACAGAAACT | CTTCACAAGCAGCAGCTAGTTCAGGACTCC | 54 |
| *rpl16* | GCTATGCTTAGTGTGTGACTCGTTG | CCCTTCATTCTTCCTCTATGTTG | 56 |
| *Xin1682f/trnK4r* | TCAGTGCTGGTTATCCAATTACAG | ATTATCTGTCAGAGGGACTAATAC | 55 |
| *trnK4f/ trnK4r* | TTTCTTAAGACTGTTCAAATTCCA | ATTGGATTTGCTGTGATA | 54 |
| *petG-trnP* | GGTCTAATTCCTCTCCCTTTGGC | GGGATGTGGCGCAGCTTGG | 55 |
| *rpL20-rpS12* | CGCCTCCGAGCTATATATCC | ATTAGAAACTCAAGACAGCCAAT | 54 |
| *ycf6-psbM* | ATGGATATAGTAAGTCTCGCTTGGGC | ATGGAAGTAAATATTCTCGCATTTATTGCT | 56 |
| *psbM-trnD^GUC^* | AGCAATAAATGCGAGAATATTTACTTCCAT | GGGATTGTAGTTCAATTGGT | 55 |
| *rpoB-trnC^GCA^* | CTACAAAATCCTTCAAATTG | CACCCGGATTTGAACTGGGG | 57 |
| *atpH-atpI* | CCAACCCAGCAGCAATAAC | TATTTACAAGTGGTATTCAAGCT | 56 |
| *ndhAx2-ndhAx1* | GGTTGACGCCACAAATTCCA | GCTCAATCGATTAGTTATGAAATACC | 55 |
| YLT1 | AAAGCCAAAGGGTCAACAGGTC | CGTCATCTTCGCAAACAGCAA | 55 |
| YLT3 | CACTTGCCCTGGATCGACTT | CCACGACCGCTGAATAGAAAC | 58 |
| YLT4 | TCGCTTATTCTGGTGTTTATCAGTGGTG | CGTCGGTGAAGACGTGTTGGTGC | 54 |
| YLT5 | TGAATGGCTCGACTAGGTAGGATAG | TGATGGGAGTTGCCGGTGTA | 53 |
| YLT7 | GAACCAGATTCCGTAAACAACATAGA | TCCCGAGCGATTGCACCA | 54 |
| YLT8 | GTACAAGGATTCCCTGAGTAAGA | AGAACCGCCTAAAGTTCCA | 56 |
| YLT9 | ATACTTCGATTCTGCCCTTCG | GGTTTGCCTTGGTATCGTGTT | 57 |
| YLT10 | AAAGTCAATCTATTCACCCGTCTA | CGGTTAGGGCAACAGTATCA | 58 |
| YLT11 | AAGTACGAAGGGGAGGGG | CGGGGATGCCGTTGAG | 56 |
| YLT12 | AATAACGGATGCCAAGAAGA | CATGCAAACTAGAAAGACCCTC | 53 |
| YLT13 | ACTACAAGTGACGGAGATACACG | GGAATAGATTCGCGGGTT | 53 |
| YLT14 | TGCTAAGAAAAGTGCTTCGGTT | AAAGAAAGGGCAGGCGTAA | 55 |
| YLT16 | GGGTCAAGGGAAAATGCC | CGATTCAGCGCCAAACA | 54 |
| YLT17 | TACTCATTAGCGAACGTCTGG | GATTTCCTGTTTGCTTACCG | 55 |
| YLT18 | GGAAGGGCCAAACAAGG | TACTGAATCGACCATAGACCAAT | 56 |
| YLT19 | CGGGAATGAATGGGAAGAT | GAGTTGGTACGGAAATGGGAT | 54 |
| YLT20 | AATCATAGGGATCTAAGCTAAGTG | CGGGCTCCATAGCAAGA | 57 |
| YLT21 | ATTTTCCGAGGGCGACA | CGTGATTAACAATTCTACCGTTC | 58 |
| YLT22 | AAAACGGACCACGCCTAAA | ATCACGGGACCACCGACAG | 56 |
| YLT23 | CTCTGCAAAGCGTAAGGGTA | TTCGTGGGTGCGGCTA | 54 |
| YLT24 | TCGAGCCGACGAGATTAG | TCTGGAGACCGAAATACCC | 57 |
| YLT25 | AGTCGAGTCTTTCGTGCCA | ACCCGTTGCCTTACCG | 53 |

Note: LX17 and LX20 were developed based on the genome sequence of *P. trichocarpa* in this study; Remaining nuclear primers followed by Du et al. (2014); plastid primers followed by Wang et al. (2015); Xin1682-F /*trnK*-4R and trnK-4F/*trnK*-R were used for sequencing locus *trnK* only.
