# Supplementary material for: Phylogenetic and Taxonomic Status Analyses of the Abaso Section from Multiple Nuclear Genes and Plastid Fragments Reveal New Insights into the North America Origin of Populus (Salicaceae)
Source: Front Plant Sci. 2017 Jan 4;7:2022. doi: 10.3389/fpls.2016.02022 (PMC5209371; doi:10.3389/fpls.2016.02022)
Supplement: Supplementary file 3 [file Table_3.DOCX]

Supplementary Material

Phylogenetic and Taxonomic Status Analyses of the Abaso section in *Populus* from Multiple Chloroplast and Nuclear DNAs reveals new insights into the North America origin of *Populus*

Xia Liu*, Zhaoshan Wang, Wenhao Shao, Zhanyang Ye, Jianguo Zhang

*** Correspondence:** Xia Liu: liuxiavip8@163.com

# Supplementary Tables

# Table S3. Characteristics of individual 23 nuclear DNA and 34 plastid fragments and combined nuclear and chloroplast fragments (excluding outgroups).

| **Nuclear locus** | **Aligned length (bp)** | **Number of variable sites (%)** | **Number of informative sites (%)** | **Model selected by AICc** |
| --- | --- | --- | --- | --- |
| DSH1 | 544 | 87 | 49 | TrN+G |
| DSH2 | 376 | 90 | 66 | HKY+I |
| DSH3 | 598 | 79 | 57 | TIM3+G |
| DSH4 | 430 | 52 | 40 | K80+I |
| DSH5 | 536 | 51 | 48 | HKY+G |
| DSH6 | 478 | 46 | 38 | TrN+I |
| DSH7 | 475 | 58 | 47 | HKY+I |
| DSH8 | 517 | 75 | 59 | HKY+I |
| DSH10 | 1109 | 175 | 111 | TPM2uf+I+G |
| DSH12 | 533 | 56 | 49 | TIM2 |
| DSH14 | 1046 | 125 | 93 | TrN+G |
| DSH15 | 876 | 92 | 78 | TPM3uf+G |
| DSH19 | 824 | 136 | 119 | TPM3uf+I |
| DSH21 | 738 | 101 | 83 | HKY+G |
| DSH22 | 872 | 159 | 137 | HKY+G |
| DSH23 | 496 | 72 | 38 | TPM1uf+G |
| DSH24 | 886 | 110 | 95 | HKY+G |
| DSH25 | 466 | 62 | 51 | HKY+I |
| DSH26 | 905 | 149 | 121 | TrN+G |
| DSH27 | 725 | 98 | 84 | HKY |
| DSH29 | 558 | 111 | 77 | TIM3+G |
| LX17 | 233 | 62 | 51 | TPM2uf |
| LX20 | 630 | 81 | 69 | TrN+G |
| Combined data | 14851 | 2127 | 1660 | TIM2+I+G |
| **Plastid locus** |  |  |  |  |
| *rpL20- rpS12（a）* | 898 | 36 | 7 | TPM2uf+I |
| *ycf6- psbM(b)* | 1498 | 45 | 12 | TVM+G |
| *psbM-trnD^GUC(c)^* | 1390 | 60 | 20 | TVM+G |
| *rpoB-trnC^GCA(d)^* | 999 | 32 | 10 | TPM1uf+I+G |
| *atpH-atpI(e* | 1137 | 29 | 16 | GTR+G |
| *ndhAx2-ndhAx1(f* | 1123 | 30 | 11 | TVM+G |
| *Rbcl(g* | 1279 | 24 | 17 | TPM3uf+I+G |
| *rpl16(h* | 1031 | 38 | 14 | TPM2uf+G |
| *trnK* | 2601 | 47 | 17 | TVM+G |
| *petG - trnP(m* | 594 | 18 | 10 | F81+I |
| *trnT-trnL(y* | 564 | 13 | 5 | TPM3uf+G |
| *trnL-trnF(q* | 1118 | 35 | 12 | TPM3uf |
| YLT1 | 1047 | 17 | 9 | TPM2uf+G |
| YLT 3 | 1954 | 29 | 7 | TVM+I |
| YLT 4 | 1956 | 21 | 8 | TIM3+I |
| YLT 5 | 1590 | 16 | 4 | TPM1uf+I |
| YLT 7 | 742 | 20 | 11 | TPM1uf+G |
| YLT 8 | 952 | 20 | 7 | TPM2uf+I |
| YLT 9 | 1069 | 12 | 2 | TPM2uf |
| YLT 10 | 833 | 20 | 6 | HKY+I |
| YLT 11 | 992 | 66 | 9 | TIM1 |
| YLT 12 | 822 | 19 | 10 | TPM1uf+I |
| YLT13 | 653 | 20 | 10 | TPM1uf+I |
| YLT14 | 814 | 15 | 7 | TVM+I |
| YLT16 | 753 | 14 | 5 | TPM1uf+I |
| YLT17 | 667 | 14 | 5 | TIM3+I |
| YLT18 | 825 | 20 | 7 | TVM+G |
| YLT19 | 848 | 9 | 2 | HKY+I |
| YLT20 | 699 | 9 | 3 | GTR |
| YLT21 | 862 | 28 | 8 | TPM2uf |
| YLT22 | 837 | 13 | 8 | TPM2uf |
| YLT23 | 1094 | 18 | 6 | TPM3uf+I |
| YLT24 | 954 | 13 | 6 | TPM2uf+I |
| YLT25 | 836 | 30 | 11 | TIM1+I |
| Combined data | 36031 | 850 | 302 | TVM+I+G |
